# Emergency Department Visits for Bicycle-Related Traumatic Brain Injuries Among Children and Adults — United States, 2009–2018

**DOI:** 10.15585/mmwr.mm7019a1

**Published:** 2021-05-14

**Authors:** Kelly Sarmiento, Tadesse Haileyesus, Dana Waltzman, Jill Daugherty

**Affiliations:** 1 National Center for Injury Prevention and Control, CDC.

Bicycling leads to the highest number of sport and recreation–related emergency department (ED) visits for traumatic brain injuries (TBIs) in the United States (*1*). Because bicycling continues to grow in popularity,* primarily among U.S. adults, examining the strategies that mitigate the risk for TBI is important. CDC analyzed data from the National Electronic Injury Surveillance System-All Injury Program (NEISS-AIP) to determine the incidence of EDs for bicycle-related TBIs during 2009–2018. An estimated 596,972 ED visits for bicycle-related TBIs occurred in the United States during the study period. Rates of ED visits were highest among adult males (aged ≥18 years) and among children and adolescents aged 10–14 years during 2009–2018. Overall, the rate of ED visits for bicycle-related TBIs decreased by approximately one half (48.7%) among children and by 5.5% among adults. As the number of persons riding bicycles increases, expansion of comprehensive bicycling safety interventions for bicyclists and drivers by states and local communities, such as interventions to increase driver compliance with traffic laws and helmet use among riders, improvements in bicycling infrastructure, and customized interventions for males and other groups at high risk might help reduce bicycle-related injuries.

NEISS-AIP, operated by the U.S. Consumer Product Safety Commission, contains annual data on patients treated in hospital EDs drawn from a nationally representative, stratified probability sample of hospitals,^†^ and weighted by the inverse probability of selection to provide national estimates. This analysis included data on bicycling-related TBIs that occurred among adults aged ≥18 years and children and adolescents (children) aged ≤17 years during 2009–2018. A case was classified as a TBI if the primary body part injured was the head and the principal diagnosis was concussion or internal organ injury. Rates of bicycle-related TBIs per 100,000 population per year were calculated by using U.S. Census Bureau population estimates as the denominator, stratified by sex and age group. Rates and 95% confidence intervals were calculated by using SAS (version 9.4; SAS Institute), accounting for sample weights and the complex survey design. Temporal trends were evaluated by applying the Joinpoint Regression Program (version 4.7.0.0; National Cancer Institute) to the annual rates. Annual percentage change was estimated for each trend segment and considered significantly different from zero for p-values <0.05. Findings were cross-validated by applying SAS complex survey software to the record-level data. This activity was reviewed by CDC and was conducted consistent with applicable federal law and CDC policy.^§^

During the 10-year study period, an estimated 596,972 ED visits involved bicycle-related TBIs (Table); most of the patients who incurred a TBI (83%) were treated and released from the ED. The rate per 100,000 population of ED visits for bicycle-related TBIs during this time decreased by 27.7%, from 18.8 in 2009 to 13.6 in 2018. The rate decrease among children aged ≤17 years (48.7%) was ninefold larger than that among adults (5.5%). From 2013 to 2018, a large overall decline occurred, resulting in an annual −9.8% decline (Figure 1).

**TABLE:** Estimated annual number and rate* of emergency department visits for all nonfatal bicycle-related traumatic brain injuries, by selected characteristics — National Electronic Injury Surveillance System–All Injury Program, United States, 2009–2018

Characteristic	2009		2018		2009–2018	
	No.† (%)	Rate (95% CI)	No.† (%)	Rate (95% CI)	No.† (%)	Rate (95% CI)
**Age group, yrs**						
0–17	28,343 (49.2)	38.2 (26.3–50.1)	14,403 (32.3)	19.6 (14.5–24.7)	**240,873 (40.3)**	**32.7 (25.2–40.1)**
0–4	2,797 (4.9)	13.8 (9.7–18.0)	986 (2.2)	—^§^	**30,614 (5.1)**	**15.3 (11.3–19.4)**
5–9	8,388 (14.6)	41.6 (25.0–58.1)	5,305 (11.9)	26.3 (17.1–35.5)	**71,763 (12.0)**	**35.2 (26.5–44.0)**
10–14	12,912 (22.4)	62.5 (43.2–81.8)	5,706 (12.8)	27.3 (18.5–36.2)	**92,316 (15.5)**	**44.6 (34.9–54.4)**
15–17	4,246 (7.4)	32.6 (15.7–49.4)	2,407 (5.4)	19.2 (11.2–27.3)	**46,180 (7.7)**	**36.4 (25.7–47.2)**
≥18	29,293 (50.8)	12.6 (5.9–19.3)	30,128 (67.7)	11.9 (6.7–17.0)	**355,869 (59.6)**	**14.6 (8.2–21.1)**
**Sex**						
Male	44,597 (77.4)	29.6 (18.5–40.6)	33,350 (74.9)	20.7 (13.3–28.1)	**448,719 (75.2)**	**28.8 (19.6–37.9)**
Female	13,038 (22.6)	8.4 (5.0–11.7)	11,181 (25.1)	6.7 (4.1–9.4)	**148,253 (24.8)**	**9.2 (5.9–12.5)**
**Disposition**						
Treated and released	48,534 (84.2)	15.8 (10.1–21.6)	36,356 (81.6)	11.1 (7.4–14.9)	**495,560 (83.0)**	**15.6 (10.7–20.6)**
Hospitalized/Transferred	7,527 (13.1)	2.5 (1.1–3.8)	7,439 (16.7)	2.3 (1.2–3.4)	**83,231 (13.9)**	**2.6 (1.4–3.9)**
Other/Unknown	1,575 (2.7)	—^§^	736 (1.7)	—^§^	**18,181 (3.0)**	—^§^
**Total^†^**	57,635 (100.0)	18.8 (11.8–25.7)	44,531 (100.0)	13.6 (8.8–18.4)	**596,972 (100.0)**	**18.8 (12.7–24.9)**

**Abbreviation**: CI = confidence interval.

^*^ Rate per 100,000 population.

^†^ Numbers might not sum to totals because of rounding.

^§^ Estimates with coefficients of variation >30%, estimated annual number of <1,200, or an unweighted count of <20 are considered unstable, and resulting rates are not reported.

**FIGURE 1:**
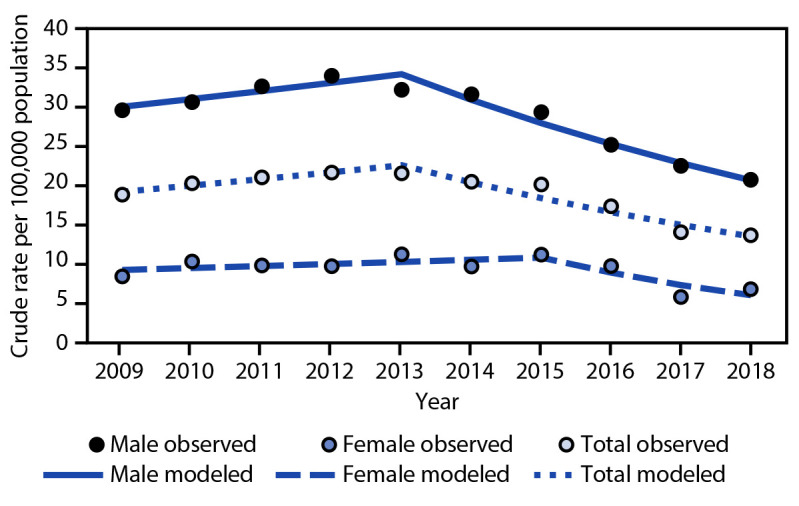
Trend in crude rates* of estimated bicycle-related traumatic brain injury emergency department visits, by sex^†^ — National Electronic Injury Surveillance System-All Injury Program, United States, 2009–2018 **Abbreviations**: APC = annual percentage change; ED = emergency department. * Crude rate per 100,000 population. Temporal trends were evaluated by applying the Joinpoint Regression Program to the annual rates. Findings were cross-validated by applying SAS complex survey software to the record-level data. † APC estimates were considered significantly different from zero for p-values <0.05. The following APC values were statistically significant: male during 2009–2013 APC = 3.30% and during 2013–2018 APC = -9.61%; total APC = -9.80%, which represents a large decline in ED visits for bicycle-related traumatic brain injuries during 2013–2018.

Across all study years, the rate per 100,000 population of ED visits for TBIs among children aged ≤17 years (32.7) was approximately twice that of adults (14.6) (Table). The rate per 100,000 population of ED visits for bicycle-related TBIs among children aged 10–14 years (44.6) was higher than that among children aged 0–4 years (15.3) and adults aged ≥18 years (14.6). Because of the limited sample size of adults, stratification by age group was not possible. The rate per 100,000 population of ED visits for bicycle-related TBIs was higher for males than for females overall (28.8 and 9.2, respectively). The estimated annual percentage change differed by sex and age group (Table) (Figure 1) (Figure 2).

**FIGURE 2:**
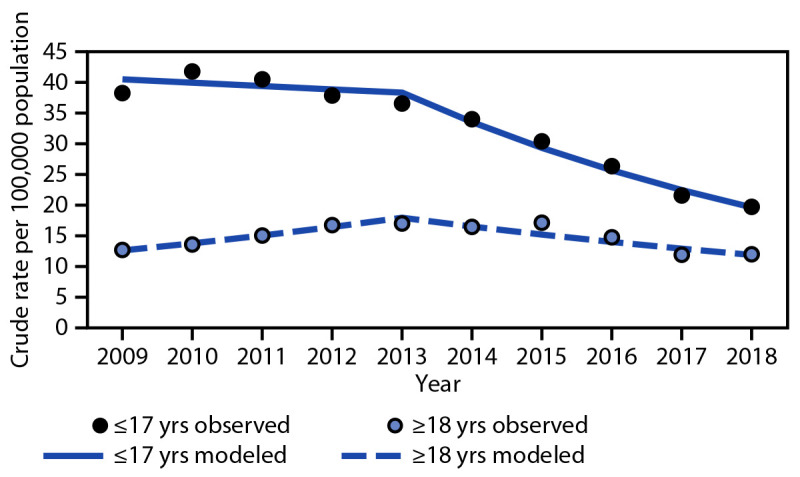
Trend in crude rates* of estimated bicycle-related traumatic brain injury emergency department visits, by age group^†^— National Electronic Injury Surveillance System-All Injury Program, United States, 2009–2018 **Abbreviation**: APC = annual percentage change. * Crude rate per 100,000 population. Temporal trends were evaluated by applying the Joinpoint Regression Program to the annual rates. Findings were cross-validated by applying SAS complex survey software to the record-level data. † APC estimates were considered significantly different from zero for p-values <0.05. The following APC values were statistically significant: children and adolescents aged ≤17 years during 2013–2018 APC = -12.57% and adults aged ≥18 years during 2009–2013 APC = 9.32% and 2013–2018 APC = -8.00%.

## Discussion

During 2009–2018, an estimated 596,972 ED visits occurred for bicycle-related TBIs in the United States. The ninefold difference in the decrease in bicycle-related TBI rates among children compared with that among adults during the study period might be associated with changes in the prevalence of bicycling (i.e., more adults bicycling, fewer children bicycling, and more bicyclists using roadways to commute to work) and with the implementation of evidence-based policies and interventions by state and local communities, many of which focus on children. The progressive decline in rates of bicycle-related TBIs that began in 2013 might be associated with increased awareness among parents about TBI and emerging research on the potential for long-term sequelae among children (*2*). Future studies should examine the reasons behind these recent improvements to help guide prevention efforts.

This study found only slight declines in the rate of ED visits for bicycle-related TBIs among adults, which is in contrast to sharp declines in rates of bicycle-related injuries and deaths among children; however, bicycle-related deaths among adults have increased in recent years (*3*). In 2018, 857 adult bicyclists died from traffic-related crashes in the United States, the highest number in two decades (*3*). This discrepancy might indicate that bicycle safety interventions have had some effect on reducing some bicycle-related TBIs among adults, but more comprehensive strategies are needed to protect cyclists from death and the most severe types of injuries (*4*). Policies that recommend the use of bicycle helmets have achieved long-term sustained helmet use rates and a 20%–55% reduction in bicycle-related head injuries, including TBIs (*4*,*5*). However, bicycle helmets are not designed to prevent a concussion, which occurs after linear and rotational forces cause extreme brain movement inside the skull (*6*). To reduce injuries and deaths, a multipronged approach that includes programmatic, environmental, behavioral, and policy interventions not solely focused on bicycle helmets might be effective (*4*). Examples of promising strategies include building or improving roads with a focus on pedestrian and bicycling safety (e.g., adding physically protected bicycle lanes and intersections), increasing compliance with traffic laws (e.g., reducing distracted driving), and increasing active bicycle lighting (e.g., equipping bicycles with lights that a bicyclist can turn on) to increase visibility of cyclists in dark conditions.^¶^

During the study period, the rate of ED visits for bicycle-related TBIs among males of all ages was three times higher than that among females. A similar disparity was found in rates of bicycle-related deaths (*3*). Expanding bicycle safety policies and associated educational efforts that include customized messages for male children and adolescents and adult males might be beneficial. Communities have had success using social marketing techniques to target bicycle injury prevention efforts to groups at risk (*7*). This might include targeted messages through media campaigns (e.g., use of social media platforms and signage in parks and public transit) about potential risk factors (e.g., distracted driving) and addressing known barriers (e.g., negative peer influence) to promote behavior change (*7*).

During the study period, most children and adults who visited an ED for a bicycle-related TBI were treated and released. Although many of these persons experienced a good recovery, some have experienced ongoing symptoms that have emotional, cognitive, behavioral, and academic sequelae (*8*). To reduce the risk for adverse outcomes, CDC has published guidelines for health care providers related to the care of children and adults with mild TBI.^**^

The findings in this report are subject to at least five limitations. First, rates of ED visits in this report likely underestimate actual rates of ED visits for bicycle-related TBIs. Many persons with TBI seek care in a primary care office or do not seek care at all (*9*). Second, because NEISS-AIP data included during the study period consisted of the principal diagnosis and primary body part recorded during the initial injury visit, some cases for which TBI was a secondary diagnosis might have been missed (such as skull fracture, which might indicate an underlying TBI). Third, this analysis did not examine differences by race/ethnicity or socioeconomic status, both of which are associated with limited bicycle safety infrastructures and an increased risk for bicycle-related injuries (*10*). Fourth, NEISS-AIP narrative descriptions do not provide detailed or consistent information about helmet use, injury circumstances (e.g., whether the injury occurred on a road or bicycle path), or about a person’s level of exposure (e.g., how often a person rides a bicycle). Finally, the available data do not allow for assessment of whether any observed differences over time in the number of bicycle-related ED visits resulted from an actual change in incidence or other reasons, such as changes in care-seeking behaviors.

Bicycling provides an important opportunity for physical activity and is a popular commuting alternative that provides both health and environmental benefits.^††^ Such interventions as increased driver compliance with traffic laws and helmet use among riders, improvements in bicycling infrastructure, and customized interventions for males and other groups at high risk might help reduce bicycle-related injuries. Thus, expanding implementation of effective bicycle safety interventions can help ensure that children and adults are afforded the benefits of bicycling while staying safe from injuries, including TBIs.

**Summary**

**What is already known about this topic?**

Although most persons treated in an emergency department (ED) for a traumatic brain injury (TBI) have a good recovery, some might experience ongoing symptoms that have emotional, cognitive, behavioral, and academic sequelae.

**What is added by this report?**

During 2009–2018, an estimated 596,972 ED visits for bicycle-related TBIs occurred in the United States. The rate of ED visits for bicycle-related TBIs decreased by approximately one half among children and adolescents aged ≤17 years and by 5.5% among adults during this time. Rates were highest among adult males and children and adolescents aged 10–14 years.

**What are the implications for public health practice?**

Expanded implementation of comprehensive bicycling safety interventions (e.g., improving compliance with traffic laws, helmet use, and bicycling infrastructure) and targeted interventions might be beneficial.

## Footnotes

*
https://www.npd.com/wps/portal/npd/us/news/press-releases/2020/cycling-industry-sales-growth-accelerates-in-april/

†
https://www.cpsc.gov/s3fs-public/2001d010-6b6.pdf

§ C.F.R. part 46.102(l)(2), 21 C.F.R. part 56; 42 U.S.C. Sect. 241(d); 5 U.S.C. Sect. 552a; 44 U.S.C. Sect. 3501 et seq.

¶
https://www.nhtsa.gov/sites/nhtsa.gov/files/documents/812478_countermeasures-that-work-a-highway-safety-countermeasures-guide-.pdf

**
https://www.cdc.gov/TraumaticBrainInjury/

††
https://www.thecommunityguide.org/findings/physical-activity-built-environment-approaches
